# Long Chain Alcohols Produced by *Trichoderma citrinoviride* Have Phagodeterrent Activity against the Bird Cherry-Oat Aphid *Rhopalosiphum padi*

**DOI:** 10.3389/fmicb.2016.00297

**Published:** 2016-03-10

**Authors:** Sonia Ganassi, Pasqualina Grazioso, Antonio De Cristofaro, Fabio Fiorentini, Maria Agnese Sabatini, Antonio Evidente, Claudio Altomare

**Affiliations:** ^1^Department of Life Science, University of Modena and Reggio EmiliaModena, Italy; ^2^Department of Agricultural, Environmental and Food Sciences, University of MoliseCampobasso, Italy; ^3^CBC (Europe) S.R.L.-Biogard DivisionCesena, Italy; ^4^Department of Chemical Sciences, University of Naples Federico IINapoli, Italy; ^5^Institute of Sciences of Food Production, National Research CouncilBari, Italy

**Keywords:** *Trichoderma*, long-chain alcohols, biocontrol, aphids, phagodeterrence

## Abstract

In this study we report the effects of fungal metabolites isolated from cultures of the fungus *Trichoderma citrinoviride* ITEM 4484 on the feeding preference of the aphid *Rhopalosiphum padi*, a major pest of cereal crops. Different phagodeterrent metabolites were purified by a combination of direct and reverse phase column chromatography and thin-layer chromatography. Chemical investigations, by spectroscopic and chemical methods, led to the identification of different long chain primary alcohols (LCOHs) of the general formula R-OH, wherein R is a long, unbranched, unsubstituted, linear aliphatic group. LCOHs have been reported as components of lepidopteran pheromone blends, but their phagodeterrent effect to aphids is herein reported for the first time. The effects of LCOHs on *R. padi* were studied by behavioral and electrophysiological bioassays. Feeding preference tests that were carried out with winged and wingless morphs of *R. padi* showed that LCOHs had high phagodeterrent activity and restrained aphids from settling on treated leaves at a concentration as low as 0.15 mM (0.036 g/l). The results of different electrophysiological analyses indicated that taste receptor neurons located on the aphid tarsomeres were involved in the LCOHs perception. Behavioral assays carried out with some commercial agrochemicals, including azadirachtin A, pyrethrum and a mineral oil-based product, in combination with 1-hexadecanol, the LCOH most abundantly produced by *T. citrinoviride* ITEM 4484, showed that these different active principles could be applied together, resulting in a useful increase of the phagodeterrent effect. The data shown indicate that these compounds can be profitably utilized for novel applications in biotechnical control of aphid pests. Furthermore, the tested LCOHs have no chiral centers and therefore can be obtained with good yield and at low cost through chemical synthesis, as well as from natural sources.

## Introduction

Aphids are insect pests of great economic importance for agriculture and represent one major cause of damage and loss of both quantity and quality of produce in horticultural, cereal and tree crops (Blackman and Eastop, 2007; Dedryver et al., 2010). Aphids use their piercing sucking mouthparts to feed on host plants causing damage by subtraction of sap, injection of saliva which has phytotoxic effects, and spread of insect-transmitted virus diseases (Hull, 2002; Ng and Perry, 2004; Katis et al., 2007). Moreover, aphids produce large amounts of honeydew, a sugary liquid waste on which sooty molds often grow hindering the plant photosynthetic capability (Dedryver et al., 2010). Various insecticides can be used to control aphids, however the intensive use of chemical insecticides has led to environmental pollution and onset of resistant populations of various aphid species (Vanlerberghe-Masutti and Guillemaud, 2007). In addition, the phasing out of some active principles and the more and more strict registration procedures for new agrochemicals have in recent times led to a progressive reduction of the number of insecticides available for aphid control (Dedryver et al., 2010; Roubos et al., 2014). Therefore, there is a growing interest in the development of innovative control strategies that may lead to a progressive reduction of chemicals, with the aim to minimize the environmental impact of pest management and improve the safety of the agro-food chain.

Signaling chemicals produced by plants (Reddy and Guerrero, 2004; Honda et al., 2010) and microorganisms (Davis et al., 2013) may evoke different behavioral responses in insects, such as attraction, repellence or aggregation stimulation. The concept of using nontoxic compounds as insect antifeedants in crop protection has grown with the demonstration of the potent feeding deterrent effect of azadirachtin and neem seed extracts to a large number of pest species (Isman, 2006). The identification of new compounds with phagodeterrent activity, which possess the capability to interfere with aphid host plant selection and host acceptance, is currently becoming of great interest for the design of innovative biotechnical strategies in control of phytophagous insects.

Different species of fungi of the genus *Trichoderma* have long been known as effective biocontrol agents of a wide range of important soil-borne and air-borne plant pathogens (Kubicek and Harman, 1998; Lorito et al., 2010). Furthemore it has been shown that some *Trichoderma* species also have a potential for biocontrol of insects (Jassim et al., 1990; Ganassi et al., 2001, 2007; Shakeri and Foster, 2007; Verma et al., 2007). In previous studies (Ganassi et al., 2002, 2007, 2009), we showed that cultures of fungal isolates belonging to four species of *Trichoderma*, namely *T. atroviride, T. citrinoviride, T. harzianum* and *T. viride*, had significant phagodeterrent activity toward different species of aphids, including *Schizaphis graminum* (Ganassi et al., 2007), one of the most important pests of cereal crops, and the polyphagous species *Myzus persicae* (Ganassi et al., 2009). Further investigations on fungal compounds produced by the isolate *T. citrinoviride* ITEM 4484 led to the isolation and structure determination by spectroscopic methods (essentially NMR and MS) of two new metabolites, namely citrantifidiene and citrantifidiol, which exhibited a potent antifeedant effect toward both winged and wingless aphid morphs (Evidente et al., 2008). Later on, two more compounds with phagodeterrent activity were isolated from the same source and identified as bislongiquinolide and dihydrotrichodimerol, two compounds belonging to the chemical family of bisorbicillinoids (Evidente et al., 2009). In this paper we report the chemical and biological characterization of one more group of phagodeterrent metabolites obtained from *T. citrinoviride* ITEM 4484, which consists of primary alcohols of the general formula R-OH, wherein R is a long, unbranched, unsubstituted linear aliphatic group. Long chain alcohols (LCOHs), varying between C10 and C18, are commonly detected especially in lepidopteran pheromone blends emitted by females to attract males (Francke and Schulz, 2010; Ando and Yamakawa, 2011), but their phagodeterrent effect to aphids and their possible use for control of this pest is herein reported for the first time. We tested the LCOHs identified in cultures of *T. citrinoviride* ITEM 4484 and other commercially available LCOHs in feeding preference assays against the bird cherry-oat aphid *Rhopalosiphum padi*, one of the most important pests of all the major cereals (Blackman and Eastop, 2000) and the most important vector of the viruses responsible of Barley Yellow Dwarf Disease (BYD) in autumn-sown cereals in Europe (Dedryver and Harrington, 2004). In addition, some LCOHs were also tested in combination with commercial agrochemicals in order to compare the effectiveness of LCOHs with that of a technology currently in use and to assess the compatibility of the active principles. Finally, we investigated the mode of perception of LCOHs by *R. padi* using a standard electroantennogram (EAG) technique and single chemosensory cell (from single tarsal sensillum) recordings.

## Materials and methods

### Fungal strain

The producing strain used in this study was isolated from soil under *Abies* sp. in Tyrol (Austria). After reisolation from a single germinated conidium, the strain was maintained in purity in the culture collection of the Institute of Sciences of Food Production with the accession number ITEM 4484. The strain was identified as belonging to the species *Trichoderma citrinoviride* on the basis of morphological characters and sequencing of a region of the nuclear rDNA comprising the two diagnostic regions ITS-1 and ITS-2 and of a fragment of the translation elongation factor gene (TEF-1α), as previously reported (Evidente et al., 2008).

### Aphids

The aphids used in tests belonged to the species *Rhopalosiphum padi*. They were reared in the laboratory on durum wheat plants (*Triticum durum*) for several generations in a thermostatic chamber at 20°C under 16/8 h day/night photoperiod to induce parthenogenesis. Trials were carried out with winged and wingless adult morphs. Winged offsprings were obtained in the laboratory by crowding.

### Production, extraction, purification and identification of metabolites with phagodeterrent activity

The fungal metabolites with phagodeterrent activity against aphids were purified from cultures of *T. citrinoviride* ITEM 4484 through a bioassay-guided chromatographic fractionation of the culture organic extract. ITEM 4484 was cultured on sterile rice kernels and the culture was processed and extracted with methanol (MeOH)-H_2_O 55:45 (v/v) and dichloromethane (CH_2_Cl_2_) and purified through chromatographic procedures previously developed and reported by Evidente et al. (2008, 2009). The organic extract was dried on sodium sulfate (Na_2_SO_4)_ and evaporated under reduced pressure, yielding 15 g of a brown oil that showed a significant phagodeterrent activity. The extract was fractionated by column chromatography (CC) using chloroform (CHCl_3_)-*iso*-propanol (*i*-PrOH) 85:15 as eluent, yielding 10 groups (A–L) of homogeneous fractions. The residue of the fraction F1 (7.5 mg) was further purified by preparative thin layer chromatography (TLC) using petroleum ether- acetone (Me_2_CO) 9:1 as eluent and yielded a mixture of LCOHs (5.4 mg) as below reported, which appeared as a homogeneous spot (*Rf* 0.29).

### Spectroscopic data of LCOHs

IR ν_max_cm^−1^ 3349, 2922, 2852, 1466; UV λ_max_ nm < 220;^1^H NMR, δ: 5.38 (m), 5.35 (m), 3.64 (t, *J* = 6.3 Hz), 2.01 (m), 1.57–1.26 (m), 0.88 (t, *J* = 6.8 Hz). ^13^C NMR, δ: 130.5 (d), 130.3 (d), 129.9 (d), 129.8 (d), 63.1 (t), 32.8–22.7 (t), 14.1 (q); EI MS (rel. int) *m/z*: 265 [M_C16_ +Na]^+^, 293 [M_C18_+Na]^+^, 291 [M_C18:1_+Na]^+^.

### Conversion of LCOHs into the corresponding esters

An aliquot of the mixture of LCOHs (0.8 mg) was dissolved in Me_2_CO (0.20 ml) at 0°C and oxidized with Jones reagent (Bowden et al., 1946) until a persistent orange color was obtained. After 10 min, all the starting compounds had reacted, as determined by TLC (eluent petroleum ether-Me_2_CO, 9:1) and the reaction was stopped by addition of *i*-PrOH. The suspension was diluted with iced H_2_O to obtain a brilliant green solution, which was extracted with ethyl acetate (EtOAc) (3 × 0.5 ml). The organic extracts were combined, dried with Na_2_SO_4_, and evaporated under reduced pressure. The residue was dissolved in MeOH (300 μl) and esterified with an ethereal solution of diazomethane (CH_2_N_2_) until a persistent yellow color was obtained. After 15 min the reaction was monitored by TLC (eluent petroleum ether-Me_2_CO, 9:1), and having all the starting compounds reacted, the reaction was stopped by evaporation under a N_2_ stream. The residue was analyzed by gas chromatography–mass spectrometry (GC-MS) as below described.

### Preparation of *cis*-9-octadecen-1-ol

Lithium aluminum tetrahydride (LiAlH_4_) (113.0 mg, 3.20 mM) was added to a solution of methyl oleate (850.0 mg, 2.87 mM) in anhydrous diethyl ether (Et_2_O) (20 ml). The reaction was stirred for 2 h at 0°C, then stopped by addition of ethyl acetate (EtOAc) to remove the excess of LiAlH_4_ and diluted with water. The aqueous solution was extracted 3 times with EtOAc and the organic layers were combined and dried with anhydrous Na_2_SO_4_. After removal of the solvent, the residue (803.5 mg) was purified by CC on silica gel, eluted with the eluent system *n*-hexane- Me_2_CO (7:3, v:v) giving 651.2 mg of *cis*-9-octadecen-1-ol with a yield of 76.6%.

### Preparation of *trans*-9-octadecen-1-ol

Methyl elaidate (888.4 mg; 3.00 mM) was converted in *trans*-9-octadecen-1-ol like previously reported for the *cis* isomer, with a yield of 75.6% (671.0 mg).

### General experimental procedures

Infrared spectroscopy (IR) spectra were recorded as glassy film on a Perkin-Elmer (Norwalk, CT, USA) Spectrum One FT-IR Spectrometer and ultraviolet (UV) spectra were taken in acetonitrile (MeCN) solution on a Perkin-Elmer Lambda 25 UV/Vis spectrophotometer. ^1^H and ^13^C nuclear magnetic resonance (NMR) spectra were recorded at 600 or 400 MHz and at 125 or 100 MHz, respectively, in deuterochlorofom (CDCl_3_), on Bruker (Karlsruhe, Germany) spectrometers. The same solvent was used as internal standard. Electrospray ionization mass spectrometry (ESI-MS) spectra were recorded on Waters Micromass Q-TOF Micro (Milford, MS, USA).

Analytical and preparative TLC was performed on silica gel, Kieselgel 60, F_254_, 0.25 and 0.5 mm respectively, (Merck, Darmstadt, Germany). The spots were visualized by spraying first with 10% sulphuric acid (H_2_SO_4_) in MeOH and then with 5% phosphomolybdic acid in ethanol (EtOH), followed by heating at 110°C for 10 min. CC was performed on silica gel column, Kieselgel 60, 0.063–0.200 mm (Merck). Standards of 1-hexadecanol and 1-octadecanol and of methyl esters of oleic and elaidic acids were purchased from Larodan Fine Chemicals (Malmö, Sweden).

The GC-MS analyses were carried out on a QP5050 Shimadzu (Kyoto, Japan) instrument equipped with a Supelcowax TM10 column, 60 m × 0.32, 0.5 μm (Supelco, Bellefonte, PA, USA). Helium was used as carrier gas at a flow rate of 2.1 ml/min, with an initial pressure of 52 Kpa. The instrument was programmed at 180°C for 15 min, increasing 10°C/min to 230°C for 20 min; the injector temperature was 270°C. The MS analysis was carried out by electron ionization (EI) at 70 eV; the interface temperature was 270°C; the source ionic temperature was 200°C; mass ranged from 40 to 450 amu; the scan rate was 0.5 scan/s.

### Behavioral assays

The phagodeterrent activity of the LCOHs 1-tetradecanol, 1-pentadecanol, 1-hexadecanol and 1-heptadecanol, were assessed by feeding preference tests on both winged and wingless morphs of *R. padi*; 1-octadecanol, *cis*-9-octadecen-1-ol, *trans*-9-octadecen-1-ol, 1-nonadecanol and 1-eicosanol, due to their limited supply, were tested only on winged morphs. For testing purposes, batches of pure LCOHs 1-tetradecanol, 1-pentadecanol, 1-heptadecanol, and 1-eicosanol were purchased by Sigma-Aldrich (St. Louis, MO, USA).

To perform the assays, the LCOHs were first solubilized in 5% (v/v) aqueous MeOH and then diluted with 5% MeOH to obtain different test concentrations. Excised wheat leaves about 5 cm in length were dipped in LCOH or control (5% MeOH) solutions for 10 s. Then, the leaves were placed on wet filter paper in 12 cm-diameter Petri dishes. Each dish contained two leaves, one treated with the test solution and one dipped in the control solution, arranged in parallel at a distance of 4 cm. Aphids were placed between the two leaves with a fine brush, and their position was recorded every hour for 8 h, starting from the initial access that began less than 1 h after leaf excision. In the tests on winged morphs the LCOHs 1-tetradecanol, 1-pentadecanol, 1-hexadecanol and 1-heptadecanol were tested at concentrations of 0.037, 0.075, 0.15, 0.3, 0.6, and 1.2 mM. Due to the limited availability of 1-octadecanol, *cis*-9-octadecen-1-ol, *trans*-9-octadecen-1-ol, 1-nonadecanol and 1-eicosanol, these compounds were tested only at the highest concentration of 1.2 mM. In tests on wingless morphs 1-tetradecanol, 1-pentadecanol, 1-hexadecanol and 1-heptadecanol were tested at concentrations of 0.075, 0.15, 0.3, 0.6, and 1.2 mM. Limitedly to winged morphs, behavioral bioassays were also carried out with three different combinations of two LCOHs chosen among the compounds that had given the best results, when assayed separately: 1-tetradecanol, 1-pentadecanol, 1-hexadecanol and 1-heptadecanol. The blends of LCOHs were made as follows: 1-tetradecanol 0.6 mM plus 1-hexadecanol 0.6 mM; 1-pentadecanol 0.6 mM plus 1-hexadecanol 0.6 mM; 1-pentadecanol 0.6 mM plus 1-heptadecanol 0.6 mM.

In addition, in order to compare the effect of LCOHs with substances that are currently in use and to assess the compatibility of active principles, behavioral assays on winged morphs were carried out with some commercial agrochemicals used in integrated pest management, and with a combination of these agrochemicals with 1-hexadecanol, the LCOH more abundantly produced by *T. citrinoviride* ITEM 4484. The tested agrochemicals were three insecticides that act by contact against a broad spectrum of insects, including aphids: viz. Pyganic®1.4 that contains natural pyrethrum, UFO®Ultra Fine Oil, containing a refined mineral oil, and NeemAzal®-T/S, containing azadirachtin A. In addition, Gondor®, a soy lecithin-based adjuvant often used in combination with the above agrochemicals was also included in the bioassay. All the agrochemicals were provided by CBC (Europe) S.R.L.-Biogard division. The dosages of the agrochemicals used for testing were those recommended for field use, that are Pyganic®1.4 2.0 ml/l; UFO® 1% (v/v); Gondor® 2.5 ml/l; NeemAzal®-T/S 2.5 ml/l. Pyganic®1.4 was tested in combination with 0.15, 0.30. 0.6, and 1.2 mM of 1-hexadecanol; UFO® was tested in combination with 0.3 and 1.2 mM of 1-hexadecanol; UFO® was tested in combination with 0.30 and 0.60 mM of 1-hexadecanol; NeemAzal®-T/S was tested in combination with 0.15 mM of 1-hexadecanol. For the assays, the agrochemicals were solubilized in distilled water and the control was distilled water. In tests carried out with 1-hexadecanol, the agrochemicals were added to solutions of 1-hexadecanol in 5% MeOH; 5% MeOH solution was used as control.

### Experimental design and statistical analysis

In the behavioral assays, 10 individuals per each treatment were tested and the experiments were repeated 12 times, except for the assays carried out on winged morphs with 1-hexadecanol 1.2 mM, 1-hexadecanol 0.075 mM, *cis*-9-octadecen-1-ol, 1.2 mM, and *trans*-9-octadecen-1-ol 1.2 mM, where 15, 21, 48, and 30 replicated experiments were run, respectively.

The raw data obtained from the feeding preference tests were analyzed by the Generalized Linear Model (GLM) for repeated measures (over time) procedure and compared by using a test of within-subjects effects. The differences between the means of the number of aphids per leaf in each of the experimental treatments and those of the number of aphids on the relevant controls over time were analyzed and adjusted by the Bonferroni test (Mukhopadhyay, 2009) for the number of comparisons.

The data of the feeding preference tests carried out with the agrochemicals and with the agrochemicals combined with different concentrations of 1-hexadecanol were utilized also to calculate the Feeding Preference Index (FPI). FPI values were calculated from the total number of aphids counted on treated (T) and control (C) leaves at hourly observations over a 8-h time interval as FPI = (C – T)/(C + T). Possible values for the index range between 1 (complete preference for control leaves) and −1 (complete preference for treated leaves), with a value equal or close to zero indicating no effect (Powell et al., 1997). For each treatment, the FPI values of the hourly observations were averaged and the data were analyzed by one-way analysis of variance (ANOVA) and Student–Newman–Keuls (SNK) multiple comparison *post-hoc* test. All the statistical analyses were done with the SPSS software for Windows, release 22.0 (SPSS, Chicago, IL, USA).

### Electrophysiological bioassays

Electrophysiological bioassays were carried out with 1-hexadecanol (1.2 mM), 1-octadecanol (1.2 mM), *cis*-9-octadecen-1-ol (1.2 mM) and *trans*-9-octadecen-1-ol (1.2 mM) as taste and antennal stimuli. Recordings by single tarsal sensilla were performed according to previous studies (Solinas et al., 2001; Ganassi et al., 2007; Evidente et al., 2009). Electrophysiological responses from the mesothoracic distal tarsomere were recorded by combining different techniques previously used to study single chemosensory sensilla (Solinas et al., 2001; Maher and Thiery, 2004; Ganassi et al., 2007). The indifferent electrode, a glass micropipette containing an Ag/AgCl silver wire (tip 4–5 μm diameter) filled with a solution of NaCl 100 mM, was inserted into the aphid prothorax. The recording electrode, a glass micropipette (tip 2 μm diameter) containing one of the test stimuli, was connected to a sensillum. Action potentials were preamplified, filtered, and recorded with commercially available electrophysiological equipment (Tasteprobe Type DTP-1, Syntech, Hilversum, The Netherlands). Taste stimuli were diluted in a 100 mM NaCl solution in 5% (v/v) MeOH; as control stimuli a 100 mM NaCl solution in distilled water or in 5% MeOH were used. Test solutions were put into the micropipette 10 s before the experiment. Single-cell recordings were carried out at 22 ± 2°C and 70 ± 10% room humidity. Electrical activity was recorded for 1 s after stimulus onset, and 5 min was allowed to elapse between presentation of successive stimuli to the same sensillum. Test and control solutions were applied in a random series on the same sensillum. Action potentials (spikes) were stored on a computer or a magnetic tape (CditII, IEC II/Type II, High Bias 70 ms EQ, position chrome, Sony, Pontonx sur l'Adour, France) by a double-channel recorder (Sony, TC-D5M) and successively analyzed with the AutoSpike 3.1 program (Syntech) on the basis of their amplitude, shape, and frequency. Sensilla that failed to respond to the tested solutions were considered not functioning and were discarded (Crnjar and Prokopy, 1982; Ganassi et al., 2007). Responses of the sensory cells were evaluated as spike frequency (spikes per second) during the first second of stimulation, 100 ms after stimulus onset. Firing frequencies were compared by means of the Student's *t*-test.

In order to verify the possible occurrence of olfactory responses, test stimuli were also tested on antennae of living aphids. Stimuli were absorbed on a piece of filter paper (1 cm^2^) inserted in a Pasteur pipette. Antennal responses were obtained by using a standard electroantennographic (EAG) technique (Ambrosi et al., 2001; De Cristofaro et al., 2004; Anfora et al., 2014), and the green leaf volatile (*Z*)-3-hexen-1-ol (25μl of a 1:10 v/v diluted solution in pure MeOH) was used as an EAG active control stimulus.

## Results

### Identification of LCOHs produced by *T. citrinoviride* item 4484

The organic extract obtained from the solid culture of *T. citrinoviride* 4484 was purified by combined CC and TLC on silica gel as described in detail in the Materials and Methods section, yielding the already known compounds citrantifidiene (acetic acid 4-acetoxy-6-hydroxy-1-(2-diydroxy-ethyl)-hexa-1,3-dienyll ester and citrantifidiol (1,2,3-trimethyl-4-(4-methylpent-3-enyl)-cyclohexane-1,3-diol (Evidente et al., 2008), trichodimerol, dihydrotrichodimerol, bislongiquinolide, and dihydrobislongiquinolide (Evidente et al., 2009) and a homogeneous oily mixture of metabolites which were identified as LCOHs as below reported.

Their IR spectrum showed bands that are typical of hydroxy and olefinic groups (Nakanishi and Solomon, 1977) while the UV spectrum consistently exhibited an end-absorption (Pretsch et al., 2000). The ^1^H and ^13^C NMR spectra of this mixture showed signals characteristic of olefinic protons and carbons [δ: 5.38 (m), 5.35 (m), and 130.5 (d), 130.3 (d), 129.9 (d), 129.8 (d)], of hydroxy and aliphatic methylene groups [3.64 (t, *J* = 6.3 Hz and 63.1 (t), 32.8–22.7 (t), and 1.57–1.26 (m)], and of terminal methyl groups [0.88 (t, *J* = 6.8 Hz) and 14.1 (q)] (Breitmaier and Voelter, 1987; Pretsch et al., 2000). Their ESI MS spectrum showed sodium clusters at *m/z* 265 [M_C16_ +Na]^+^, 293 [M_C18_+Na]^+^, 291 [M_C18:1_+Na]^+^ consistent with the presence of C_16_ and C_18_ saturated and C_18_ unsaturated fatty alcohols.

The LCOHs were identified by GC-MS analysis as follows. They were first converted into the corresponding acids by oxidization with Jones reagent and then converted in the corresponding methyl esters by reaction with an ethereal solution of diazomethane. The esters so obtained were subjected to GC-MS analysis, as reported in the experimental section, in comparison with chemical reference standards. They were identified as the methyl esters of hexadecanoic and octadecanoic acids and the *cis* and *trans* stereoisomers of the 9-octadecenoic acid.

Therefore, the alcohols produced by *T. citrinoviride* and purified as a mixture as above reported were identified as 1-hexadecanol, 1-octadecanol and *cis*- and *trans*-9-octadecen-1-ol. These latter compounds were also prepared with high yields in one step from the corresponding commercially available esters (methyl oleate and methyl elaidate, respectively) by reduction with LiAlH_4_.

### Behavioral assays

Data of behavioral assays were analyzed with the GLM repeated measures procedure. This analysis assesses whether the interaction between either test conditions (treated or untreated) and the changes over the time of the number of aphids that visited the treated leaves or the control leaves is statistically significant. No time × treatment interaction effect indicates that the number of aphids per treated leaf and the number of aphids per control leaf did not change over time. In our bioassays a significant time × treatment interaction effect at a given concentration indicated that the number of aphids counted on control leaves increased over the time of the trial. The Bonferroni test was used to assess whether the average number of aphids on treated leaves was significantly smaller than that on corresponding control leaves, over time, indicating a phagodeterrent effect of the test solution.

The results of phagodeterrent effect of LCOHs on *R. padi* winged morphs are shown in Table 1.

**Table 1:** **Effect of long-chain alcohols at different concentrations on feeding preference of *Rhopalosiphum padi* winged morph**.

**Test compound**	**Concentration (mM)**	**GLM (time × treatment)^a^**		**Bonferroni test^b^**	
				**Mean treatment**	**Mean control**
1-Tetradecanol	1.2	*F*_(7, 154)_ = 4.230	*P* < 0.0001	1.938±0.322	6.271±0.322^**^
	0.6	*F*_(7, 154)_ = 2.318	*P* < 0.05	2.365±0.264	5.292±0.264^**^
	0.3	*F*_(7, 154)_ = 1.186	*P* > 0.05	3.271±0.217	5.552±0.217^**^
	0.15	*F*_(7, 154)_ = 10.72	*P* < 0.0001	2.375±0.370	5.980±0.370^**^
	0.075	*F*_(7, 154)_ = 5.173	*P* < 0.0001	3.500±0.384	4.958±0.384^*^
	0.037	*F*_(7, 154)_ = 1.210	*P* > 0.05	3.969±0.414	5.146±0.414n.s.
1-Pentadecanol	1.2	*F*_(7, 154)_ = 2.354	*P* < 0.05	1.365±0.301	7.615±0.301^**^
	0.6	*F*_(7, 154)_ = 11.052	*P* < 0.0001	2.385±0.237	6.188±0.237^**^
	0.3	*F*_(7, 154)_ = 4.686	*P* < 0.0001	2.271±0.283	6.292±0.283^**^
	0.15	*F*_(7, 154)_ = 4.265	*P* < 0.0001	3.406±0.405	5.083±0.405^**^
	0.075	*F*_(7, 154)_ = 1.576	*P* > 0.05	3.073±0.335	5.594±0.335^**^
	0.037	*F*_(7, 154)_ = 0.674	*P* > 0.05	4.954±0.383	4.854±0.383n.s.
1-Hexadecanol	1.2	*F*_(7, 196)_ = 5.488	*P* < 0.01	1.308±0.315	7.717±0.315^**^
	0.6	*F*_(7, 154)_ = 4.367	*P* < 0.01	2.281±0.371	6.531±0.371^**^
	0.3	*F*_(7, 154)_ = 1.336	*P* > 0.05	2.635±0.323	6.635±0.323^**^
	0.15	*F*_(7, 154)_ = 2.837	*P* > 0.05	3.938±0.429	5.479±0.429^*^
	0.075	*F*_(7, 280)_ = 0.791	*P* > 0.05	4.024±0.349	5.113±0.349^*^
	0.037	*F*_(7, 154)_ = 1.390	*P* > 0.05	3.719±0.352	4.125±0.352n.s.
1-Heptadecanol	1.2	*F*_(7, 154)_ = 6.940	*P* < 0.0001	1.094±0.294	6.427±0.294^**^
	0.6	*F*_(7, 154)_ = 0.863	*P* > 0.05	2.177±0.298	5.240±0.298^**^
	0.3	*F*_(7, 154)_ = 1.265	*P* > 0.05	3.063±0.532	5.448±0.532^**^
	0.15	*F*_(7, 154)_ = 2.892	*P* < 0.01	3.240±0.410	5.677±0.410^**^
	0.075	*F*_(7, 154)_ = 2.654	*P* < 0.05	4.250±0.373	5.406±0.373^*^
	0.037	*F*_(7, 154)_ = 1.912	*P* > 0.05	3.854±0.274	4.417±0.274n.s.
1-Octadecanol	1.2	*F*_(7, 154)_ = 1.012	*P* > 0.05	2.469±0.333	5.927±0.333^**^
*cis-*9-Octadecen-1-ol	1.2	*F*_(7, 658)_ = 9.093	*P* < 0.0001	3.518±0.235	5.826±0.235^**^
*trans-*9-Octadecen-1-ol	1.2	*F*_(7, 406)_ = 1.557	*P* > 0.05	3.938±0.291	5.067±0.291^**^
1-Nonadecanol	1.2	*F*_(7, 154)_ = 1.573	*P* > 0.05	3.427±0.391	4.792±0.391^*^
1-Eicosanol	1.2	*F*_(7, 154)_ = 5.048	*P* < 0.01	3.042±0.303	5.073±0.303^**^

a *Values of P < 0.05 for GLM indicate that the interaction between either test conditions (treated or control) and the change over time was statistically significant. Values of P > 0.05 for GLM indicate that the interaction between either test conditions (treated or control) and the change over time was not statistically significant*.

b The average number of aphids on treated leaf and the number of aphids on the control leaf over the duration of the assay were analyzed and adjusted with Bonferroni test for the number of comparisons. In each treatment, significant difference between the means are indicated with

* (P < 0.05) or with

** *(P < 0.01), ns indicates a not significant difference*.

#### 1-tetradecanol

The GLM analysis of the data revealed time × treatment interaction effects at the concentrations 1.2, 0.6, 0.15, and 0.075 mM. On the contrary, at the concentrations 0.3 and 0.037 mM no time × treatment interaction effect was found. Over time, the average number of aphids on leaves treated with 1-tetradecanol at the concentrations of 1.2, 0.6, 0.3, 0.15, and 0.075 mM was significantly smaller than the number of aphids on the relevant control leaves, but no significant difference was found at the concentration of 0.037 mM (Bonferroni test).

#### 1-pentadecanol

The GLM analysis of the data revealed time × treatment interaction effects at the concentrations 1.2, 0.6, 0.3, and 0.15 mM, while no time × treatment interaction effect was found at the concentrations of 0.075 and of 0.037 mM. The average number of aphids on leaves treated with 1-pentadecanol at all the tested concentrations, except 0.037 mM, was significantly smaller than that on corresponding control leaves over time (Bonferroni test).

#### 1-hexadecanol

The GLM analysis revealed time × treatment interaction effects at the concentrations of 1.2 and 0.6 mM. No time × treatment interaction effects were found with concentrations of 0.3, 0.15, 0.075, and 0.037 mM. The average number of aphids on leaves treated with 1-hexadecanol 1.2, 0.6, 0.3, 0.15, or 0.075 mM was significantly smaller than that on control leaves over time, while at concentration 0.037 mM it was not significantly different from control (Bonferroni test).

#### 1-heptadecanol

Time × treatment interaction effect was found at the concentrations 1.2, 0.15, and 0.075 mM, while no time × treatment interaction effects were found with 1-heptadecanol 0.6, 0.3, and 0.037 mM. The average number of aphids on leaves treated with 1-heptadecanol at all the concentrations tested, except 0.037 mM, was significantly smaller than that on control leaves over time (Bonferroni test).

#### 1-octadecanol

The GLM analysis of the data obtained in tests carried out with 1-octadecanol tested at concentration 1.2 mM, revealed no time × treatment interaction effects. The Bonferroni test showed that the average number of aphids on treated leaves was significantly smaller than that on corresponding control leaves over time.

#### *cis*-9-octadecen-1-ol

The GLM analysis of the data obtained in tests carried out with *cis*-9-octadecen-1-ol 1.2 mM revealed time × treatment interaction effects. The Bonferroni test showed that the average number of aphids on treated leaves was significantly smaller than that on corresponding control leaves over time.

#### *trans*-9-octadecen-1-ol

The GLM analysis revealed no time × treatment interaction effects of *trans*-9-octadecen-1-ol applied at the concentration 1.2 mM. The Bonferroni test showed that the average number of aphids on treated leaves was significantly smaller than that on corresponding control leaves over time.

#### 1-nonadecanol

No time × treatment interaction effect was found for 1-nonadecanol tested at 1.2 mM. The Bonferroni test showed that the average number of aphids on treated leaves was significantly smaller than that on control over time.

#### 1-eicosanol

The GLM analysis of the data obtained in tests carried out with 1-eicosanol 1.2 mM revealed time × treatment interaction effects. The Bonferroni test showed that the average number of aphids on treated leaves was significantly smaller than the number of aphids on corresponding control leaves over time.

The results of phagodeterrent effect of LCOHs on *R. padi* wingless morphs are shown in Table 2.

**Table 2:** **Effect of long-chain alcohols at different concentrations on feeding preference of *Rhopalosiphum padi* wingless morph**.

**Test compound**	**Concentration (mM)**	**GLM (time × treatment)^a^**		**Bonferroni test^b^**	
				**Mean treatment**	**Mean control**
1-Tetradecanol	1.2	*F*_(7, 154)_ = 4.527	*P* < 0.01	2.208±0.259	7.104±0.259^**^
	0.6	*F*_(7, 154)_ = 1.953	*P* > 0.05	2.813±0.308	5.594±0.308^**^
	0.3	*F*_(7, 154)_ = 9.320	*P* < 0.01	3.583±0.239	5.271±0.239^**^
	0.15	*F*_(7, 154)_ = 3.430	*P* < 0.01	3.542±0.253	5.177±0.253^**^
	0.075	*F*_(7, 154)_ = 2.566	*P* < 0.01	4.083±0.557	4.667±0.557n.s.
1-Pentadecanol	1.2	*F*_(7, 154)_ = 11.926	*P* < 0.01	2.313±0.321	6.719±0.321^**^
	0.6	*F*_(7, 154)_ = 6.624	*P* < 0.01	2.125±0.378	5.958±0.378^**^
	0.3	*F*_(7, 154)_ = 0.940	*P* > 0.05	2.563±0.163	6.104±0.163^**^
	0.15	*F*_(7, 154)_ = 3.382	*P* < 0.05	3.083±0.301	5.188±0.301^**^
	0.075	*F*_(7, 154)_ = 0.0931	*P* > 0.05	4.625±0.412	4.333±0.412n.s.
1-Hexadecanol	1.2	*F*_(7, 154)_ = 8.041	*P* < 0.01	2.698±0.291	5.354±0.291^**^
	0.6	*F*_(7, 154)_ = 2.660	*P* < 0.05	2.604±0.347	5.385±0.347^**^
	0.3	*F*_(7, 154)_ = 0.499	*P* > 0.05	3.167±0.460	5.479±0.460^**^
	0.15	*F*_(7, 154)_ = 1.192	*P* > 0.05	3.906±0.421	5.240±0.421^*^
	0.075	*F*_(7, 154)_ = 2.017	*P* > 0.05	4.135±0.430	4.646±0.430n.s.
1-Heptadecanol	1.2	*F*_(7, 154)_ = 1.168	*P* < 0.01	2.188±0.247	6.561±0.247^**^
	0.6	*F*_(7, 154)_ = 11.885	*P* < 0.01	2.354±0.231	6.094±0.231^**^
	0.3	*F*_(7, 154)_ = 3.647	*P* < 0.01	3.771±0.393	4.979±0.393^*^
	0.15	*F*_(7, 154)_ = 0.744	*P* > 0.05	3.958±0.377	5.344±0.377^*^
	0.075	*F*_(7, 154)_ = 0.996	*P* > 0.05	4.375±0.280	4.750±0.280n.s.

a *Values of P < 0.05 for GLM indicate that the interaction between either test conditions (treated or control) and the change over time was statistically significant. Values of P > 0.05 for GLM indicate that the interaction between either test conditions (treated or control) and the change over time was not statistically significant*.

b The average number of aphids on treated leaf and the number of aphids on the control leaf over the duration of the assay were analyzed and adjusted with Bonferroni test for the number of comparisons. In each treatment, significant difference between the means are indicated with

* (P < 0.05) or with

** *(P < 0.01), ns indicates a not significant difference*.

#### 1-tetradecanol

The GLM analysis of the data obtained in tests carried out with 1-tetradecanol 1.2, 0.3, 0.15, and 0.075 mM revealed time × treatment interaction effects. No time × treatment interaction effect was found with the concentration 0.6 mM. The average number of aphids on leaves treated with 1-tetradecanol at the concentrations 1.2, 0.6, 0.3, and 0.15 mM was significantly smaller than the number of aphids on corresponding control leaves, over time, while no significant difference was found with 0.075 mM (Bonferroni test).

#### 1-pentadecanol

Time × treatment interaction effect was found for 1-pentadecanol applied at 1.2, 0.6, and 0.15 mM, but not at 0.3 and 0.075 mM. The average number of aphids on leaves treated with 1-pentadecanol 1.2, 0.6, 0.3, and 0.15 mM, but not 0.075 mM, was significantly smaller than that on the relevant control leaves, over time (Bonferroni test).

#### 1-hexadecanol

The GLM analysis of the data of 1-hexadecanol applied at 1.2 and 0.6 mM revealed time × treatment interaction effects. No time × treatment interaction effects were found at the concentrations 0.3, 0.15, and 0.075 mM. The Bonferroni test showed that the average number of aphids on leaves treated with 1-hexadecanol 1.2, 0.6, 0.3, and 0.15 mM, but not with 1-hexadecanol 0.075 mM, was significantly smaller than the number of aphids on corresponding control leaves over time.

#### 1-heptadecanol

Time × treatment interaction effects were found for 1-heptadecanol at 1.2, 0.6, and 0.3 mM, but not at 0.15, and 0.075 mM (GLM analysis). The average number of aphids on treated leaves treated with 1-heptadecanol at the concentrations 1.2, 0.6, 0.3, and 0.15 mM was significantly smaller than that on corresponding control leaves, while at a concentration of 0.075 mM the effect was not statistically significant.

Phagodeterrent effect of LCOHs blends are reported in Table 3. The blends tested on winged morphs of *R. padi* were indicated in the proper Materials and Methods section. In all the combinations, the GLM analysis revealed time × treatment interaction effects. The Bonferroni test revealed that the average number of aphids on treated leaves was significantly smaller than the number of aphids on corresponding control leaves, respectively, over time (Table 3).

**Table 3:** **Effect of long chain alcohols blends on feeding preference of *Rhopalosiphum padi* winged morph**.

**Test blend**	**GLM (time × treatment)^a^**		**Bonferroni test^b^**	
			**Mean treatment**	**Mean control**
1-Tetradecanol 0.6 mM+ 1-Hexadecanol 0.6 mM	*F*_(7, 154)_ = 5.703	*P* < 0.05	1.448±0.851	7.698±0.851^**^
1-Pentadecanol 0.6 mM + 1-Hexadecanol 0.6 mM	*F*_(7, 154)_ = 9.247	*P* < 0.05	1.979±0.367	7.240±0.367^**^
1-Pentadecanol 0.6 mM + 1-Heptadecanol 0.6 mM	*F*_(7, 154)_ = 5.554	*P* < 0.05	1.854±0.346	7.115±0.346^**^

a *Values of P < 0.05 for GLM indicate that the interaction between either test conditions (treated or control) and the change over time was statistically significant*.

b The average number of aphids on treated leaf and the number of aphids on control leaf over the duration of the assay were analyzed and adjusted with Bonferroni test for the number of comparisons. In each treatment, significant difference between the means are indicated with

** *(P < 0.01)*.

Phagodeterrent effect of hexadecanol in combination with agrochemicals are reported in Table 4. The GLM analysis of the data obtained in experiments carried out with the test solutions as described in the Materials and Methods section, revealed time × treatment interaction effects (Table 4). For the treatments Gondor® 2.5 ml/l, NeemAzal®-T/S 2.5 ml/l and NeemAzal®-T/S 2.5 ml/l plus 1-hexadecanol 0.15 mM, the GLM analysis showed that the interaction between the number of aphids on treated leaf and the number of aphids on control leaf did not change over time (Table 4). The Bonferroni test revealed that in all the above treatments the average number of aphids on treated leaves was significantly smaller than the number of aphids on the control leaves, indicating that all the agrochemicals and all the combinations of agrochemicals and different concentrations of 1-hexadecanol that were tested had phagodeterrent effect (Table 4).

**Table 4:** **Effect of 1-hexadecanol (He) in combination with different agrochemicals on feeding preference of *Rhopalosiphum padi* winged morph**.

**Test solution**^**a**^	**GLM (time** × **treatment)**^**b**^		**Bonferroni test**^**c**^	
			**Mean treatment**	**Mean control**
Pyganic®1.4 (Py)	*F*_(7, 154)_ = 4.341	*P* < 0.01	1.042±0.310	6.469±0.310^**^
Py + He 1.2 mM	*F*_(7, 238)_ = 3.202	*P* < 0.01	0.486±0.197	6.625±0.197^**^
Py + He 0.6 mM	*F*_(7, 238)_ = 10.787	*P* < 0.01	0.347±0.251	7.444±0.251^**^
Py + He 0.3 mM	*F*_(7, 238)_ = 16.337	*P* < 0.01	0.319±0.219	6.819±0.219^**^
Py + He 0.15 mM	*F*_(7, 196)_ = 3.241	*P* < 0.01	0.396±0.664	6.264±0.542^**^
UFO® (UFO 1%)	*F*_(7, 154)_ = 2.573	*P* < 0.05	1.25±0.365	6.27±0.365^**^
UFO + He 1.2 mM	*F*_(7, 196)_ = 13.118	*P* < 0.01	0.367±0.247	8.250±0.247^**^
UFO + He 0.3 mM	*F*_(7, 196)_ = 7.137	*P* < 0.01	0.683±0.259	7.108±0.259^**^
Gondor® (Go)	*F*_(7, 196)_ = 0.396	*P* > 0.05	2.16±0.243	6.28±0.243^**^
Go + He 0.6 mM	*F*_(7, 196)_ = 9.471	*P* < 0.01	0.692±0.184	7.933±0.184^**^
Go + He 0.3 mM	*F*_(7, 196)_ = 6.307	*P* < 0.01	1.267±0.232	6.592±0.232^**^
NeemAzal®-T/S (Ne)	*F*_(7, 154)_ = 0.525	*P* > 0.05	3.76±0.709	5.33±0.709^*^
Ne + He 0.15 mM	*F*_(7, 154)_ = 0.182	*P* > 0.05	1.281±0.184	2.875±0.184^**^

a *The agrochemicals were tested at the dosages recommended by the manufacturers: Pyganic® 1.4, 2.0 ml/l; UFO®, 1% (v./v.); NeemAzal®-T/S, 2.5 ml/l. Gondor® was tested at 2.5 ml/l*.

b *Values of P < 0.05 for GLM indicate that the interaction between either test conditions (treated or control) and the change over time was statistically significant. Values of P > 0.05 for GLM indicate that the interaction between either test conditions (treated or control) and the change over time was not statistically significant*.

c The average number of aphids on treated leaf and the number of aphids on the control leaf over the duration of the assay were analyzed and adjusted with Bonferroni test for the number of comparisons. In each treatment, significant difference between the means are indicated with

* (P < 0.05) or with

** *(P < 0.01), ns indicates a not significant difference*.

**Figure 1:**
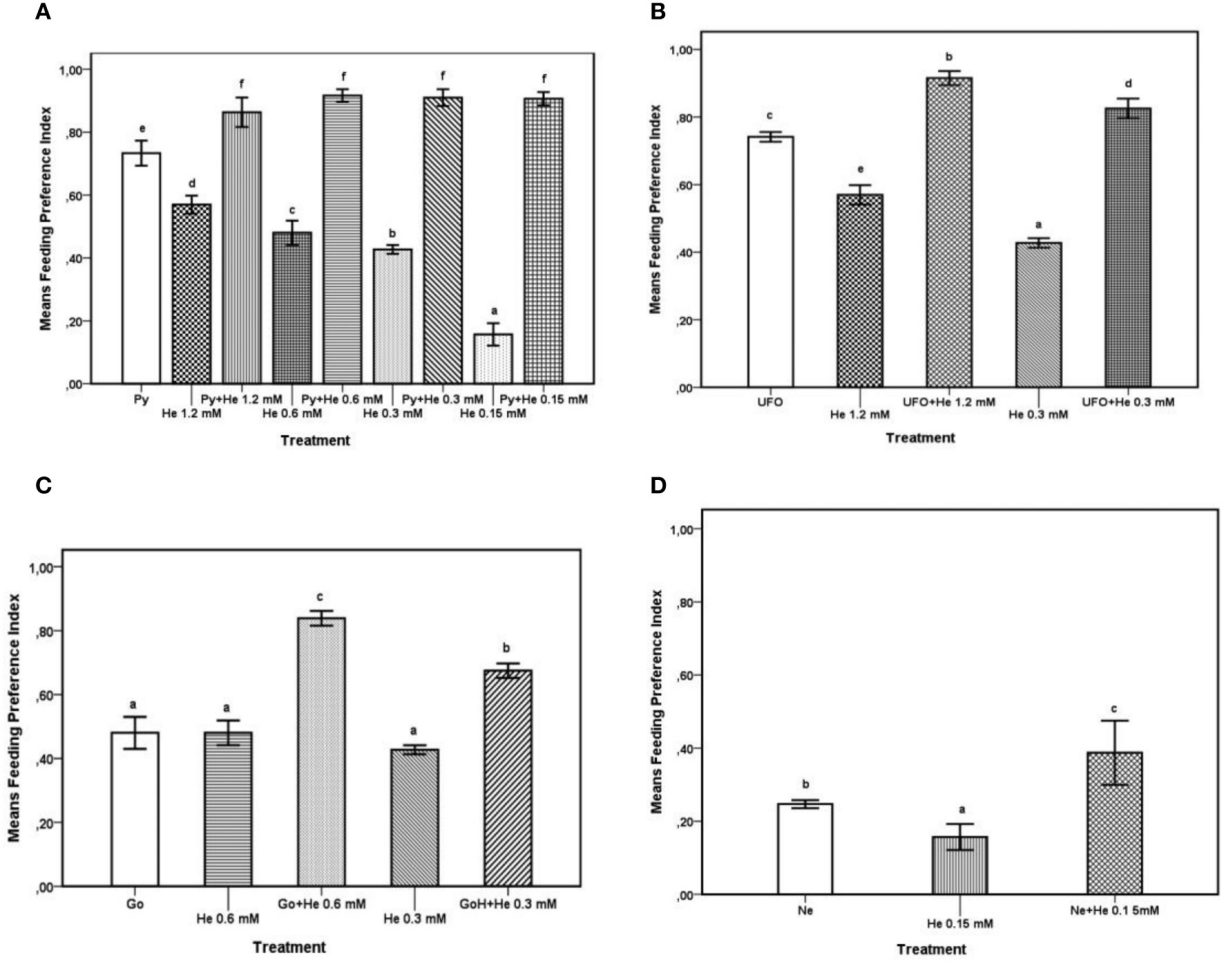
**Feeding preference test on winged morphs**. For each treatment, the Feeding Preference Index (FPI) value is the mean of 8 h records. Possible values for the index range between 1 (complete phagodeterrent activity) and −1 (complete preference for treated leaves), with a value equal or close to zero indicating no effect. Bars with different letters are significantly different (*P* < 0.05) according to the SNK test. **(A)** FPI values of Pyganic®1.4 at the dose 2.0 ml/l (Py,), 1-hexadecanol (He) 1.2, 0.6, 0.3, 015 mM, and blends of Py plus He at different concentrations (1.2, 0.6, 0.3, 015 mM). **(B)** FPI values of UFO® at the dose 1% (UFO®), 1-hexadecanol (He) 1.2 and 0.3 mM, and blends of UFO® with either 1.2 or 0.3 mM of He. **(C)** FPI values of Gondor®, applied at the dose of 2.5 ml/l (Go), 1-hexadecanol (He) 0.6 and 0.3 mM and blends of Go with 0.6 or 0.3 mM of 1-hexadecanol. **(D)** FPI values of NeemAzal®-T/S at the dose 2.5 ml/l (Ne), 1-hexadecanol (He) 0.15 mM, and a blend of Ne with 0.15 mM of hexadecanol.

The FPI values obtained from feeding preference tests carried out with the agrochemicals and with the agrochemicals combined with different concentrations of 1-hexadecanol were subjected to ANOVA and SNK test for multiple comparisons; results are reported in Figures 1A–D. The results of comparison of FPI values of Pyganic®1.4 at the dosage for field application (2.0 ml/ml), different doses of 1-hexadecanol, and a combination thereof are shown in Figure 1A. The addition of 1-hexadecanol in a range of concentrations from 0.15 to 1.2 mM to Pyganic®1.4, significantly (*P* < 0.05) increased the phagodeterrent effect of the agrochemical. The addition of 1-hexadecanol increased the efficacy of Pyganic®1.4 regardless of the concentration. The combination of UFO® 1% with either 1.2 or 0.3 mM of 1-hexadecanol significantly increased the phagodeterrent activity of the agrochemical (*P* < 0.05; Figure 1B). Gondor® was tested at 2.5 ml/l in combination with 0.6 and 0.3.M mM of 1-hexadecanol, and these mixtures increased the phagodeterrent activity, as determined by the FPI (Figure 1C). The combination of NeemAzal®-T/S with 0.15 mM of 1-hexadecanol significantly (*P* < 0.05) increased the phagodeterrent effect of the agrochemical (Figure 1D).

### Electrophysiological bioassays

EAG responses showed that the tested LCOHs were not able to stimulate significantly the olfactory sensilla of either winged or wingless morphs of *R. padi* in respect of the solvent (MeOH). The antennae, on the contrary, were stimulated by the control stimulus, the green leaf volatile *cis*-3-hexen-1-ol that elicited significant responses (0.6 ± 0.2 mV).

Electrophysiological bioassays carried out on sensilla of the mesothoracic distal tarsomere revealed that the applications of either control solutions, i.e., NaCl (100 mM) or NaCl (100 mM) in 5% MeOH, evoked action potential frequencies similar to those obtained in resting activity. Single taste tarsal cells of winged and wingless morphs were significantly stimulated by 1-hexadecanol (1.2 mM) and 1-octadecanol (1.2 mM) and highly significantly stimulated by *cis*-9-octadecen-1-ol (1.2 mM) and *trans*-9-octadecen-1-ol (1.2 mM) with a significant increase (*P* < 0.01) of the frequency of action potentials over the controls (Table 5). Results of single chemosensory cell recordings indicate that the structures involved in the LCOHs perception are taste cells located on the aphid tarsomeres.

**Table 5:** **Action potentials (spikes/s ± SD) recorded by single taste tarsal cells of winged (*n* = 6) and wingless (*n* = 8) morph of *Rhopalosiphum padi* on stimulation with long-chain alcohols produced by *T. citrinoviride* ITEM 4484**.

**Tested stimulus**	**Winged morphs**	**Wingless morphs**
Resting activity	12.4 ± 2.2	18.3 ± 3.2
NaCl (100 mM)	12.6 ± 1.8	18.3 ± 3.2
NaCl (100 mM) + MeOH (5%)	12.5 ± 2.2	18.5 ± 3.4
1-Hexadecanol (1.2 mM)	28.6 ± 4.3^*^	31.4 ± 4.6^*^
1-Octadecanol (1.2 mM)	30.8 ± 4.2^*^	35.2 ± 4.2^*^
*cis*-9-Octadecen-1-ol (1.2 mM)	54.5 ± 6.4^**^	56.7 ± 7.4^**^
*trans-*9-Octadecen-1-ol (1.2 mM)	58.5 ± 8.2^**^	61.4 ± 9.3^**^

*On the same column, significant difference between resting activity and tested stimulus (t-test;

* *P < 0.05*,

** *P < 0.01)*.

## Discussion

LCOHs are known signaling molecules in different insect groups, especially in Lepidoptera where they are part of pheromone blends (Francke and Schulz, 2010; Ando and Yamakawa, 2011). In particular, primary alcohols and their derivatives (mainly acetates and aldehydes) with a long straight chain (C10–C18) are commonly detected in pheromone-gland extracts of lepidopteran females and have been found to explicate an attractive action toward males (Ando and Yamakawa, 2011). Our results show that the LCOHs obtained from the isolate *T. citrinoviride* ITEM 4484 and LCOHs chemically related to them also have a strong phagodeterrent activity toward winged and wingless morphs of *R. padi* and interestingly, in several trials, the number of aphids on the control leaves increases over time.

Behavioral assays carried out with some insecticides in combination with 1-hexadecanol, the LCOH most abundantly produced by *T. citrinoviride* ITEM 4484, showed that 1-hexadecanol is compatible with different commercial active principles and the phagodeterrent effect of the mixture increases significantly. The agrochemicals Pyganic®1.4 and NeemAzal®-T/S are natural compound-based insecticides utilized in integrated pest management. Their phagodeterrence is due to the presence of pyrethrum and azadirachtin A, respectively. Pyrethrum and its analogs pyrethrins are widely used as insecticides in agricultural, public health, and domestic applications (Wesseling et al., 2001; Ray and Fry, 2006) and have also a repellent effect toward different species of insects (Baumler and Potter, 2007; Patel et al., 2012; Prota et al., 2014). Azadirachtin blocks the synthesis and release of molting hormones from the prothoracic gland, leading to incomplete ecdysis in immature insects and to sterility in adult females and is a potent antifeedant compound to many insects species (Isman, 2006; Gahukar, 2014). Mineral oils, like the main active principle of UFO® (Ultra Fine Oil), have also been reported to exhibit repellent action against some insect pests (Mounts et al., 1988; Yang et al., 2010). In our experiments, 1-hexadecanol increased significantly the phagodeterrent effect of Pyganic®1.4, NeemAzal®-T/S and Gondor®, applied at the dosage recommended for field application. The increasing effect of 1-hexadecanol in combination with these products was already significant at a dose as low as 0.15 mM (0.036 g/l).

The EAG study carried out with the LCOHs produced by *T. citrinoviride* ITEM 4484 (hexadecanol, octadecanol, *cis*-9-octadecenol and *trans*-9-octadecenol) showed that these LCOHs compounds are not able to stimulate the antennal olfactory receptor neurons of *R. padi*. The results of single chemosensory cell recordings indicate that taste cells located on the aphid tarsomeres are involved in the perception of the tested LCOHs. In previous papers we showed that such taste cells were also involved in the perception of other or unidentified metabolites produced by *Trichoderma* species, including *T. citrinoviride*, by different aphid species (Ganassi et al., 2007; Evidente et al., 2009).

Studies on *Cydia pomonella* pheromone composition reported that the LCOH 1-tetradecanol is among the 5 components of the female effluvia (El-Sayed et al., 1999; Witzgall et al., 2001). Ebbinghaus et al. (1998) reported that some *C. pomonella* sensilla auricillica contained olfactory receptor neurons, one of which responded to minor components, including 1-tetradecanol and 1-hexadecanol. 1-Hexadecanol and 1-octadecanol are parts of *Heliothis virescens* male pheromone blend (Teal and Tumlinson, 1989) and 1-hexadecanol was discovered in *Helicoverpa armigera* sex pheromone blend along with many other components (Witzgall et al., 2010). Gas chromatography-flame ionization detection coupled with electroantennographic detection analyses have put in evidence that 1-hexadecanol in the extract of *H. armigera* female sex pheromone gland was electrophysiologically active to male antennae (Zhang et al., 2012). Hexadecanol and tetradecanol were also identified in species-specific marking pheromones, produced by males of different species of the genus *Bombus*, to attract conspecific females for mating (Urbanova et al., 2004). Keeling et al. (2003) showed that hexadecanol is a synergistic component of the honeybee queen pheromone blend to attract a retinue of workers. Jin et al. (2006) stated that *Locusta migratoria* odorant binding protein1 (LmigOBP1) displayed significant specificity to linear aliphatic alcohols and ketones of approximately 15-carbon chain length, and in particular LmigOBP1 interacted preferentially with 1-pentadecanol and 2-pentadecanone. Qiao et al. (2013) investigated the ligand-binding properties of two *Bombyx mori* chemosensory proteins (CSPs) and showed that CSP2 had a high affinity for most of aliphatic and aromatic compounds tested and the best ligands were those with 12–16 carbon atoms. Oviposition deterrence evoked by resistant cultivars of *Zea mays* toward *Chilo partellus* was attributed partly to larger quantities of 1-nonadecanol and 1-heptadecanol, compared to susceptible cultivars (Varshney et al., 2003).

Long chain aliphatic alcohols are employed for a wide variety of industrial and commercial uses, relying on their lubricating, emollient, solubilizing, or emulsifying properties. They can be found in some pharmaceutical products, agrochemical formulations, household cleaning, and personal care products (Modler et al., 2004; Veenstra et al., 2009). As far as the risk of LCOHs to human health is concerned, representative compounds from this category have been extensively tested to assess their toxicity. Acute oral toxicity data in species other than rat are limited, but confirm the very low acute toxicity of these alcohols to humans (OECD, 2006). Inhalation of vapors of LCOHs in the range C6–C22 at levels up to the saturated vapor pressure is unlikely to be associated with significant toxicity; also, these chemicals are not regarded as allergy sensitizers (OECD, 2006). Given the widespread use in cosmetic industry and the relatively low numbers of reported cases of allergy, it can be concluded that LCOHs have a very low allergenic potency (Veenstra et al., 2009). The available data on eye irritation indicate that the LCOHs that may induce different levels of irritation are those with chain length comprised between C6–C11, while the eye irritation potential of LCOHs with a chain length of C12 and above is minimal (OECD, 2006). Studies on the effects of these chemicals on mammals other than humans do not show evidence of genetic toxicity or detrimental effects to the reproductive system or the developing organism (Veenstra et al., 2009).

In conclusion, the LCOHs isolated from cultures of *T. citrinoviride* ITEM 4484 and other LCOHs structurally related to them proved to function as signaling molecules that can modify the aphid feeding preferences. Based on these results, LCOHs might be useful for control of aphid pests and prevention of both direct damage caused by sap subtraction and indirect damage caused by transmission and spread of plant virus diseases. The LCOHs proved to be compatible with other agrochemicals used in organic farming, such as pyrethrum, azadirachtin A and mineral oil, and improve their efficacy. In our preliminary field trials (data not shown) the addition of 1-hexadecanol to Pyganic®1.4 showed promising results and encourages more extensive studies in the field.

The practical use of these compounds for crop protection is expected to have a low environmental impact and, due to their mode of action, not to be a threat to beneficial insects. The low toxicity and allergenicity of LCOHs let foresee a facilitated and faster evaluation process for registration purposes. An additional advantage represented by these metabolites is that they have no chiral centers and therefore can be obtained in good yields through chemical synthesis, besides than from natural sources. All together these features encourage further studies aiming at the implementation of novel agrotechnical products for aphid control and crop protection based on LCOHs. Part of the results herein presented are the subject of an international patent application (Sabatini et al., 2012) concerning “Phagodeterrent compounds of fungal origin” (PCT/IB2012/052383).

## Author contributions

AC, isolation, identification, molecular characterization, and mass production of the producing strain. Experiments planning and design; SG, aphid behavioral bioassays and electrophysiological studies. Experiments planning and design; PG, Statistical analyses of feeding preference data; AC, electrophysiological studies. FF, Studies on the combined use of long shan alcohols and agrochemicals. MS, aphid behavioral studies and bioassays. AE, Chemical extraction of fungal cultures, fractionation and purification of active metabolites, characterization of long chain alcohols by spectroscopic, chemical and GC methods.

## Funding

Work of SG was partly supported by the project “Aficontrol” (prot. N. 352/08) of the program “Spinner 2013” of Emilia Romagna Region (Italy); work of AE was partly supported by “P.Q.R. Campania FERS 2007/2013—Bio Industrial Processes (BIP),” CUP B25C13000290007.

### Conflict of interest statement

The authors declare that the research was conducted in the absence of any commercial or financial relationships that could be construed as a potential conflict of interest.
